# Modeling Skin Injury from Hot Spills on Clothing

**DOI:** 10.3390/ijerph14111374

**Published:** 2017-11-11

**Authors:** Torgrim Log

**Affiliations:** Department of Engineering, Western Norway University of Applied Sciences, Haugesund 5528, Norway; torgrim.log@hvl.no; Tel.: +47-900-500-01

**Keywords:** scalding, fabric, damage integral, thermal injury, numerical modeling

## Abstract

The present work analyzes scald burns from hot beverages, such as coffee and tea, spilled on the lap, i.e., an incident that may occur in daily life. The Pennes bioheat equation is solved numerically for small spills wetting the clothing, i.e., the fabric prevents the spilled liquid from draining away. Temperatures are analyzed in the wetted fabric and the skin layers and the resulting skin injury is calculated based on the basal layer temperature. Parameters influencing burn severity, such as clothing thickness, liquid temperature, removal of fabric and thermal effects of post scald water cooling are analyzed. The fabric cools the water some but represents a threat since the entrapped water results in a prolonged heat supply. The liquid temperature turned out to be the most important injury parameter, where liquid temperature of about 80–85 °C seems to be a limit for developing superficial partial-thickness burns in the present minimum case, i.e., where the liquid just wets the fabric. Spilling water in excess of just wetting the fabric, more severe burns will develop at lower liquid temperatures due to the prolonged heat supply. Higher liquid temperatures will nearly instantly develop more severe burns. It is demonstrated that removal of the clothing within the first seconds after the spill may significantly reduce the scalding severity. The general advice is therefore to avoid excessive heating of beverages and, if the beverage is spilled, to quickly remove the wetted clothing. Prolonged tempered water cooling is advised to improve the healing processes.

## 1. Introduction

Burn injuries are of major concern worldwide. On a global scale, approximately 265,000 deaths occur every year due to burns. Skin burns are a common injury in all societies, as a result of hot liquid spills, contact with hot surfaces and exposure to hot gases or heat radiation. In the country of Bangladesh, around 173,000 children under 18 sustain a burn injury annually [1]. In the USA, burn injuries every year result in close to half a million patients seeking medical treatment at hospital emergency departments. This number comes in addition to burns treated at clinics, community health centers, or by private medical offices [2]. It is also difficult to heal severe burns and much research is focused on burn injury treatment [3,4,5]. Better knowledge about the development of thermal skin injury is therefore much appreciated.

Systematic research into the field of thermal skin injury started just after World War II. A series of seminal studies on this topic was published in *The American Journal of Pathology*. These studies included heat transport to and through, porcine skin with temperature recordings [6]. The importance of time and surface temperature in causing cutaneous burns [7] was also studied, in addition to the pathology and pathogenesis of cutaneous burns on pigs [8]. The degree of burns development depends on the temperature and exposure time and several researchers refer to temperature >44 °C causing burns [9,10]. For hot fluid scalding, other researchers refer to 43 °C as the onset of injury [11]. Skin simulators have also been built for studying heat transfer and comparing the developed models to recorded “skin” temperatures during controlled heat flux exposure [12]. An Arrhenius type of damage development is often assumed, i.e., the damage increases considerably with excess temperature.

Pain receptors are located at a depth of approximately 0.1 mm and the pain temperature threshold is 44.8 °C [13,14]. This is above the temperature for slow burns development but as burns usually involve much higher basal layer temperatures, the pain signal is a warning about excessive heating. In burn scenarios, the skin heating is often quite instantaneous, i.e., the damage develops even though the victim is warned by the ongoing process. Scalding by hot beverages is an example of this nearly instant skin heating well above the pain threshold temperature. The best way to reduce the scald risk is to prevent potential heat exposure. However, as hot beverages and other hot liquids is a part of most, if not all, cultures scalds happen.

It is known to the research and medical communities that removing the heat source and cooling the affected area, is very beneficial. Based on a recent statistical analysis of patient journals for scalding injuries involving Chinese congee (high viscosity rice soup), Lau et al. [15] documented that prompt removal of clothing after congee scalding reduced post-burn morbidity. They did, however, comment that this straightforward act is sometimes not being made in a timely manner due to embarrassment in e.g., restaurants, etc., as well as wrong information about the importance of heat source removal.

In special situations, skin heat exposure may be studied using analytic solutions [16]. In most scalding situations, however, the analytic solutions are too limited for covering the complicated boundary conditions. Numerical solutions are much more flexible and allows for analyzing the situation with realistic thermal properties of the involved skin layers, blood perfusion and metabolic heat production. The modelling in the present work is therefore based on numerically solving the Fourier-type heat transfer equation, which has recently been used successfully by other researchers [17,18,19].

The purpose of the present work is to analyze the temperature development and skin injury for spills of hot liquids to clothing covering the skin and preventing water run-off. The influence of spill temperature, removal of involved fabric, water cooling, etc., is studied and the injury for these cases is calculated based on the temperature of the basal layer. Spills on the thigh are used as an example. The paper is unique in analyzing the whole process including heat transport in the wet and hot clothing and in the skin layers. Skin temperatures are presented for selected cases as well as the injury development. Some final conclusions are drawn regarding the most influential parameters and actions that may limit the associated skin injury.

The paper starts with explaining research on burns and burn modelling (Section 1). Then the theory of heat transfer, blood perfusion and metabolic heat production is presented as well as the damage integral and numerical modeling parameters (Section 2). Then, the findings are presented (Section 3) before the modeling and the results are discussed (Section 4) followed by the conclusions (Section 5). A strong motivation for completing and publishing this work is to provide information about possible mitigation measures should the scalding happen to any of the readers or to their next in kind.

## 2. Heat Transport and Damage Integral

### 2.1. Heat Transport Modelling

According to Fourier’s law, the heat conducted in a solid may be described by a linear relationship between the temperature gradient and the heat flux:

(1) $${\dot{q}}_{k}^{\prime \prime }=-k\cdot \nabla T\;\left(W/m^{2}\right)$$

where k (W/m·K) is the thermal conductivity of the solid, i.e., the skin. The general heat balance for bioheat transfer may be expressed by Pennes bioheat equation:

(2) $$\rho C\frac{\partial T}{\partial t}=-\nabla \cdot {\dot{q}}_{k}^{\prime \prime }+W_{b}\rho_{b}C_{b}\left(T_{b}-T\right)+Q_{met}+Q_{ext}\;\left(W/m^{3}\right)$$

where ρ (kg/m^3^) is the skin density, C (J/kg·K) is the skin specific heat, t (s) is the time, $W_{b}$ (m^3^/m^3^·s) is the blood perfusion rate, $\rho_{b}$ (kg/m^3^) is the density of blood, $C_{b}$ (J/kg·K) is the specific heat of blood, $T_{b}$ (K) is the temperature of the supplied blood, $Q_{met}$ (W/m^3^) is the metabolic heat production and $Q_{ext}$ (W/m^3^) is heat supplied from an external heat source.

During short duration heat exposures, Ng and Chua [20] concluded that the blood perfusion did not significantly influence the extent of burns. This confirmed the opinion of Lipkin et al. [21] that about 20 s is needed for the skin to increase the blood flow. Recently, Fu et al. [9] came to a similar conclusion. However, since up to 30 s heat exposure is analyzed in the present study, blood perfusion and metabolic heat production is included. The models presented by Rai and Rai [22] for blood perfusion and metabolic heat production were therefore incorporated in the bioheat equation, i.e., Equation (2).

Since water wets skin completely, the external heat source, i.e., the wet and hot fabric, is assumed to be in perfect contact with the skin surface. Since the diameter of the thigh is much larger than the heat penetration depth, the system may be considered as a one-dimensional, i.e., flat, body surface. This allows for studying heat flow in one dimension, i.e., dependent on the depth (*x*-dimension) only. And since a moderate temperature increase is studied in the present work, it is assumed that the thermal skin properties are independent of temperature.

**Figure 1:**
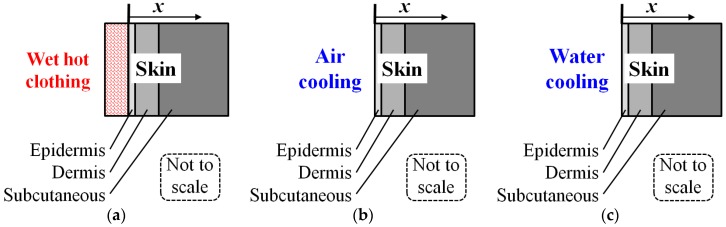
Principle sketch of the one-dimensional heat transfer system: (**a**) During wet and hot clothing skin contact; (**b**) after fabric removal; and (**c**) during final water cooling. (*x* indicates the skin depths, i.e., for simplicity, the clothing is located at *x* < 0.)

The heat transfer model is shown in Figure 1a for the heating, Figure 1b for removed fabric and in Figure 1c for the possible final cooling by tempered water.

In the present work, it is assumed that wetting of the clothing with hot liquid happens instantaneously at t = 0 and that the temperature of the wet fabric at that moment is uniform. At t > 0, heat is conducted from the wet and hot fabric into the skin. This case is then compared to cases where the wet and hot fabric is suddenly removed after a defined delay. For some cases, the skin is also finally exposed to running water for efficient post scald cooling.

When the fabric is at the skin, the convective heat loss from the outside fabric surface at temperature $T_{f,s}$ (°C) to ambient air at temperature $T_{\mathrm{air}}$ (°C) is given by:

(3) $${\dot{q}}_{\mathrm{air}}^{\prime \prime }=h_{\mathrm{air}}\cdot \left(T_{f,s}-T_{\mathrm{air}}\right)\;\left(W/m^{2}\right)$$

where $h_{\mathrm{air}}$ (W/m^2^·K) is the actual surface to air convective heat transfer coefficient. Equation (3) is also applied to the cooling of the skin when exposed to ambient air after removal of the clothing, by substituting the fabric surface temperature ($T_{f,s}$) with the skin surface temperature, $T_{s,s}$ (°C). For the cases where the skin is cooled by running water at temperature $T_{w}$ (°C) and convective heat transfer coefficient $h_{w}$ (W/m^2^·K), the heat loss is similarly given by:

(4) $${\dot{q}}_{w}^{\prime \prime }=h_{w}\left(T_{s,s}-T_{w}\right)\;\left(W/m^{2}\right)$$

In the present work $h_{\mathrm{air}}$ and $h_{w}$ were estimated to 10 W/m^2^·K and 600 W/m^2^·K, respectively.

Solving Equation (2) numerically opens for including successive boundary conditions for heat transport to the skin by (i) conduction from the wet hot fabric in the first period, $t_{f}$ (s), of contact with the fabric, (ii) air cooling and finally (iii) cooling by running water after $t_{w}$ (s). For simplicity, the initial skin temperature at t=0 was set to 37 °C [11] for all depths 0 ≤ x ≤ Δ, where Δ (m) is the domain size including the skin layers of Table 1. For the period of contact with the wet and hot fabric of thickness L (m), the boundary condition to the air surface at x=−L is given by:

$$k\frac{\partial T}{\partial x}=h_{\mathrm{air}}\left(T_{\mathrm{air}}-T\left(-L,t\right)\right)\;\mathrm{for}\;0<t\leq t_{f}$$

At time $t_{f}$, the wet and hot fabric is instantaneously removed and the skin surface at x=0 is then exposed to the ambient air, with the following boundary condition:

$$k\frac{\partial T}{\partial x}=h_{\mathrm{air}}\left(T_{\mathrm{air}}-T\left(0,t\right)\right)\;\mathrm{for}\;t_{f}<t\leq t_{w}$$

At time $t_{w}$, the final cooling of the skin surface in running water is started, with the following boundary condition:

$$k\frac{\partial T}{\partial x}=h_{w}\left(T_{w}-T\left(0,t\right)\right)\;\mathrm{for}\;t>t_{w}$$

The boundary condition for the inner surface, i.e., at x=Δ, is for simplicity given by the contact with an adiabatic surface, i.e.,:

$$k\frac{\partial T}{\partial x}=0\;\mathrm{for}\;\mathrm{all}\;t\;\mathrm{at}\;x=\Delta$$

The domain size (depth of the skin) must be large enough to limit any influence of the finite dimensions. A depth ∆ > $2\sqrt{at}$ is normally required to minimize the influence of the reflectance heat wave of a limited domain [23], where t (s) is the time and a (m^2^/s) is the thermal diffusivity given by:

(5) $$a=\frac{k}{\rho C}\;(m^{2}/s)$$

The muscle layer was also included in the calculation domain, though not strictly necessary according to the ∆ > $2\sqrt{at}$ criterion.

The literature values for skin layer thicknesses vary, probably as a result of large individual differences. A comprehensive summary of skin thicknesses and properties as they relate to burns are provided by Johnson et al. [11]. They do, however, not give any values for the thigh epidermis thickness. Their reported values vary from those reported by Millington and Wilkinson [24], who give a thigh epidermis thickness of 54.3 μm. Since the epidermis thickness also probably varies with the location on the thigh, i.e., probably thicker on the outwards facing surface and thinner on the inward facing surfaces, 40 μm, 50 μm and 60 μm will be used in the present work. In a study regarding scalding on the shoulder, Abraham et al. [19] used 80 μm for the epidermal thickness. This value is therefore also tested for checking the influence of a quite thick epidermis layer. 

In order to achieve numerical stability, the Fourier number must satisfy:

(6) $$Fo=a\cdot \frac{∆t}{∆x^{2}}<0.5$$

where ∆t (s) is the numerical integration time interval and ∆x (m) is the layer thickness. A computer program was written in the C++ language to solve the involved numerical equations for the respective boundary conditions. For the numerical integration ∆x = 10 μm and ∆t = 2 × 10^−4^ s were used to comply with Equation (5) ensuring numerical stability for the skin layer of the highest thermal diffusivity, i.e., the muscle layer.

### 2.2. Burn Modelling

Burns may be classified as first-degree burns, second-degree and third-degree burns due to the burn severity. Burns may, however, also be classified into one of four categories: (1) *Superficial* (S) burns confined to the epidermal layer and characterized by slight edema and fast healing; (2) *Superficial partial-thickness* (SP) burns that extend into the outer part of the dermal layer and result in moderate edema but little or no scarring (typically less than 1 mm burn depth); (3) *Deep partial-thickness* (DP) burns that extend well into the dermal layer and are slow to heal (typically 1 mm or greater burn depth) and results in hypertrophic scarring; and (4) *full-thickness* (FT) burns that extend through the entire dermis requiring skin grafting (burn depths typically greater than 2 mm) [19]. Typically, S corresponds to commonly used term first-degree burns, SP and DP to second-degree burns and FT to third-degree burns. 

The cell injury due to excessive skin temperatures and protein breakdown, where collagen is one of the main proteins involved can be calculated to evaluate the burn severity at certain depths. The cell injury is expressed by the damage index Ω [25]:

(7) $$\Omega \left(\tau \right)=-\ln\left(\frac{C_{\tau }}{C_{0}}\right)$$

where $C_{0}$ and $C_{\tau }$ respectively represent the number of undamaged cells prior to and after the heat exposure. A damage index of 0.1 indicates that 90% of the cells are undamaged while a damage index of 1.0 indicates that only 36% of the cells are still undamaged. The rate of the developing skin damage can be calculated using the activation energy based model developed by Henriques [26]:

(8) $$\frac{\partial \Omega }{\partial t}=P\;\exp\left(-\frac{\Delta E}{RT}\right)$$

where P (1/s) is the pre-exponential frequency factor, ΔE (J/mol) is the activation energy, R (8.314 J/mol·K) is the molar gas constant and T (K) is the absolute temperature. Literature data for these damage parameters vary significantly [11]. In the present case, as in [11], where hot water represented the heat source, it is appropriate to use the original data presented by Henriques and Moritz [6,26], i.e., P = 3.1 × 10^98^ 1/s and ΔE = 6.28 × 10^8^ J/mol. 

The total burn damage is obtained by integrating over the time interval where the basal layer temperature is ≥43.0 °C:

(9) $$\Omega =\int_{0}^{t}P\;\exp\left(-\frac{\Delta E}{\mathrm{RT}}\right)\mathrm{dt}$$

Since the damage index is calculated as an integral, it is often referred to as the damage integral. Ye and He [27] report a damage integral Ω = 0.53 at the basal layer as the limit for superficial burns and Ω = 1.0 as the limit for superficial partial-thickness burns. Comparing the calculated damage integral with these values was done to discuss skin injury for different exposure situations. Numerical integration of Equation (9) was in the present work also done in the previously mentioned C++ program.

### 2.3. Wet Fabric Temperature

The temperature of the wet fabric is a function of the initial temperatures of fabric and water as well as the respective masses and specific heats. These parameters are also to some extent temperature dependent. When soaking wet as a result of spilling e.g., hot tea, the thermal heat capacity of the water will dominate the fabric heat capacity as it wets the cotton fibers and removes most, if not all, the previously contained air. The thermal conductivity of the soaked cotton is taken as the thermal conductivity of water since water dominates the wet fabric. At a representative temperature of 330 K, the thermal conductivity of water is 0.65 W/m·K [28].

The density of cotton clothing may vary considerably. In the present study, a density of 200 kg/m^3^ is used [29]. The fabric thickness may also vary considerably. In the present work, it is assumed to be 1.0 mm thick for the base case.

Before spilling hot liquid, the mean fabric temperature is set to 30 °C. The temperature of the wet cotton fabric just after spilling is given by instantaneously filling water to the voids of the fabric replacing the previously thermally insulation air. Given the thermal properties in Table 2, the temperature, $T_{0,wf}$ (°C), of the wet fabric after an instantaneous liquid spill, is given by:

(10) $$T_{0,wf}=\left(\rho_{f}C_{f}T_{f}+\rho_{w}C_{w}T_{w}\right)/\left(C_{f}T_{f}+C_{w}T_{w})\;({}^{\circ}C\right)$$

where $\rho_{w}$ (kg/m^3^) is the actual water density (concentration) within the fabric. The subscript f and w represent fabric and water, respectively. It should be noted that the resulting temperature $T_{0,wf}$ given by Equation (10) is dominated by the hot water, e.g., filling the fabric pores with water at 85 °C results in a wet fabric temperature of 80.9 °C. For looser woven cotton, the resulting temperature would be even higher. The estimated properties of the soaked cotton fabric given in Table 2 is used for modelling the heat transport from the hot wet fabric to the skin surface (until the fabric is removed from the skin surface).

### 2.4. The Spill Situations to be Analyzed

A situation with 50 μm epidermis thickness, 1 mm fabric thickness and a spill of water at 85 °C fully wetting the fabric, which was not removed, was defined as a base case for burn injury calculation. Then, the injury development was calculated for situations where the wet and hot fabric was removed. Injury development was also calculated for selected situations where epidermal thickness, fabric thickness (fully wetted) and water temperature were varied. This is explained in more details in the Results section.

## 3. Results

**Figure 2:**
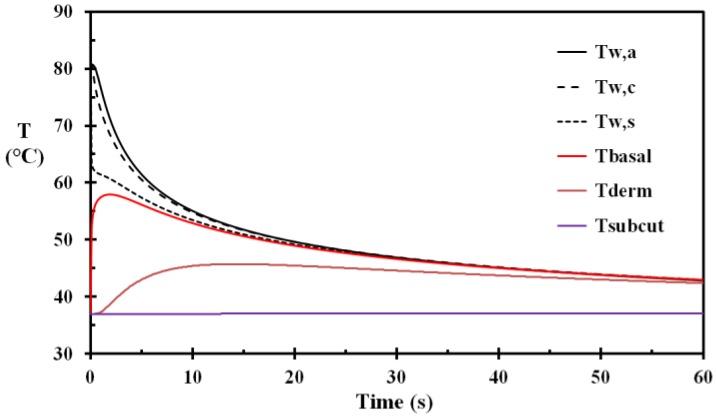
Temperatures for Case A versus time: T_w,a_: outer fabric layer; T_w,c_: center fabric layer; T_w,s_: skin surface fabric layer; T_basal_: basal layer temperature; T_derm_: center of the dermis; and T_subcut_: center of the subcutaneous layer. (The fabric was not removed.)

**Figure 3:**
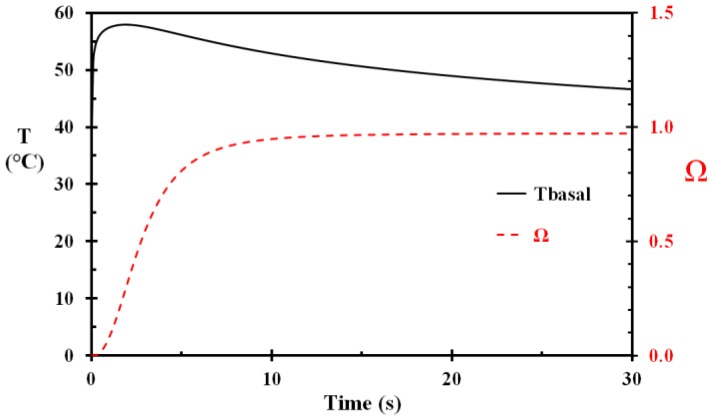
A close-up of the basal layer temperature (T_basal_) and the corresponding damage integral (Ω) as a function of time for Case A. (The fabric was not removed.)

The first modeling (Case A) was performed with an epidermis thickness of 50 mm and water at 85 °C spilled to the fabric, according to Equation (10) instantly giving a wet fabric of temperature 80.9 °C. The temperature versus time for selected depths, including the fabric, is shown in Figure 2. A close-up of the basal layer temperature and the corresponding injury development, without removing the wet and hot fabric, is shown in Figure 3. It is evident from Figure 3 that the damage integral develops rapidly, i.e., during the first few seconds of the heat exposure. The conclusion that the first 5 s period is critical for injury development is also supported by other researchers, e.g., [17].

Results from different cases, e.g., removal of the fabric, application of cooling water, different epidermal thickness, etc., are presented in Table 3 to study the influence on calculated skin injury. Removal of the hot fabric at 5 s and water cooling from 10 s reduced the damage integral from 0.973 to 0.829, i.e., Case B. The water cooling at 10 s did, however, not significantly reduce the damage integral, as seen when comparing Case B and Case D, with damage integrals 0.829 and 0.830, respectively. The similar conclusion may be drawn when comparing Case C, Case E and Case F, where the early removing of the hot fabric results in the reduction in skin injury, while the cooling with water only 0.1 s after fabric removal did not significantly reduce the damage integral. Case C does, however, clearly demonstrate the benefit of removing the hot fabric at 2.5 s resulting in a damage integral reduction from 0.973 to 0.475, i.e., from a superficial partial thickness burn to a superficial burn.

**Figure 4:**
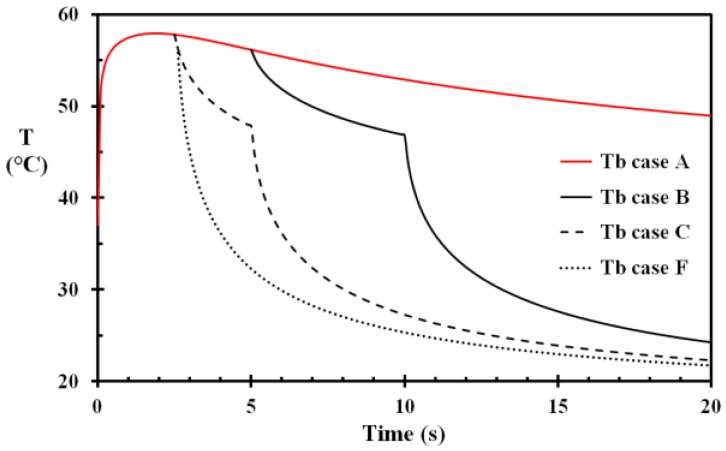
Basal layer temperature as a function of time for Case A, B, C and F. Case A: fabric not removed and no water cooling. Case B: fabric removed at 5 s and water cooling applied from 10 s. Case C: fabric removed at 2.5 s and water cooling applied from 5 s. Case F: fabric removed at 2.5 s and water cooling applied from 2.6 s.

**Figure 5:**
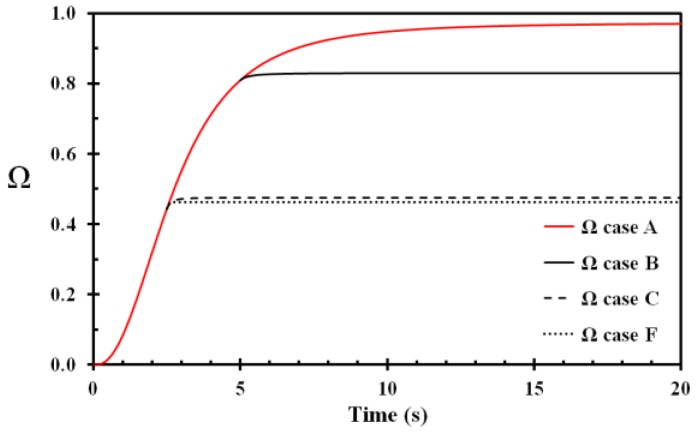
Damage integral (Ω) as a function of time for the basal layer for Case A, B, C and F. Case A: fabric not removed and no water cooling. Case B: fabric removed at 5 s and water cooling applied from 10 s. Case C: fabric removed at 2.5 s and water cooling applied from 5 s. Case F: fabric removed at 2.5 s and water cooling applied from 2.6 s.

**Figure 6:**
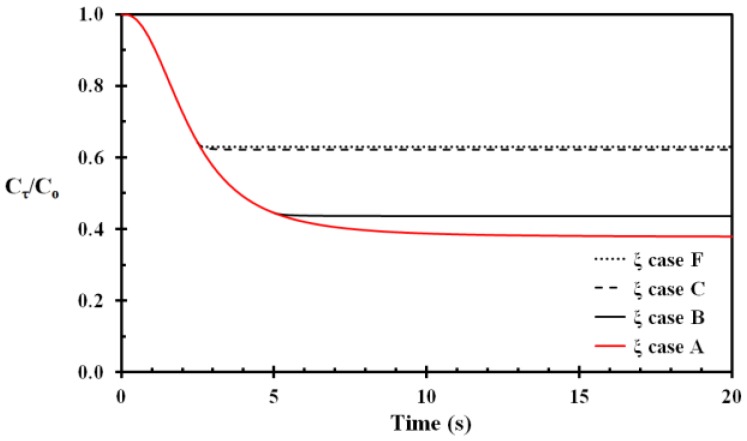
The fraction of basal layer cells undamaged after heat exposure as a function of time for Case A, B, C and F. Case A: fabric not removed and no water cooling. Case B: fabric removed at 5 s and water cooling applied from 10 s. Case C: fabric removed at 2.5 s and water cooling applied from 5 s. Case F: fabric removed at 2.5 s and water cooling applied from 2.6 s.

The temperature development of the basal layer for Case A, Case B, Case C and Case F is shown in Figure 4. The damage integral and the fraction of undamaged cells for these cases are shown Figure 5 and Figure 6, respectively. The benefit of prompt removing the wet and hot fabric with respect to injury development is clearly demonstrated in Figure 5 and Figure 6. For this type of skin injury, removing the hot fabric nearly instantly halts the injury development. It is also seen that applying tempered water 0.1 s after fabric removal contributes only to a minor extent in damage reduction. In a real situation, it is also quite unrealistic that removing the fabric as early as 2.5 s is followed 0.1 s later by tempered water application to the scalded area. The instant thermal effects of the water cooling may therefore be quite limited.

The results presented in Table 2 indicate that a water temperature of 85 °C in several situations results in superficial partial thickness burns, even though the fabric is just soaked and there is no excess water supply. A water temperature of 80 °C gives margin also to a superficial burn for the just soaked fabric thicknesses studied in the present work.

In the base case, i.e., Case A, the epidermal thickness was 50 μm. Reducing the thickness to 40 μm (Case G) increased the damage integral from 0.973 to 1.209. Larger epidermal thicknesses, e.g., 60 μm and 80 μm, reduced the damage integral to 0.792 and 0.542, respectively. It may therefore be concluded that the thickness of the epidermis is very important in hot liquid spills in the setting studied in the present work. This was also the concluded by [30].

Increasing the fabric thickness by 10% resulted in an increase in damage integral from 0.973 to 1.290 (Case J). Reducing the fabric thickness by 10% resulted in a decrease in damage integral from 0.973 to 0.706 (Case K). It should be noted that in the present study, increasing or decreasing the fabric thickness implies that more or less hot water was added to instantly wet the fabric.

When the temperature of the spilled water was increased from 85 °C to 90 °C the instant wet fabric temperature according to Equation (10) became 85.6 °C. This resulted in a significant increase in damage integral, i.e., from 0.973 to 4.267. Reducing the temperature of the spilled water from 85 °C to 80 °C resulted in a decrease in damage integral from 0.973 to 0.227. This corresponds to a decrease in injury severity from a superficial partial thickness burn to below the threshold recognized for superficial burns. This demonstrates that the temperature of the spilled water is very important when it comes to potential scald consequences. This agrees with the results obtained by other researchers, e.g., [18].

The results show that variations in conditions, such as spilled liquid temperature, fabric thickness and epidermal thickness, greatly influence the calculated damage integrals. 

## 4. Discussion

The numerical model, which has also been used by other researchers, e.g., [31,32,33,34], was developed further to include skin contact with fabric suddenly soaked with water mimicking spilled beverages. The temperature was modeled in both the hot fabric and the skin layers. The model allowed for changing boundary conditions and relevant parameters thereby providing valuable information about the skin temperature development. The model provided knowledge regarding the potential factors influencing the skald injury, such as fast removal of the wet hot fabric and subsequent tempered water application to the affected skin.

For simplicity, the initial skin temperature was set to 37 °C. Though this may be somewhat conservative, the clothing will result in quite uniform skin temperatures. This conservative skin temperature has also been used by others [11]. The benefit of this simplification is that one does not need to introduce an initial skin temperature gradient.

It was clearly demonstrated that the water temperature is of great importance regarding development of scald injury. It was also demonstrated that fast removal of the heat source was the most important factor after the scalding had happened. The fabric should be removed as early as possible, i.e., within the first seconds, to significantly limit the injury development. The finding that the first few seconds is critical is supported also by other researcher, e.g., [17]. Following the fabric removal, even unrealistic fast tempered water application to the heated skin did only give a minor injury reduction. It should, however, be mentioned that there are several research studies concluding that prolonged tempered water cooling of burned skin significantly reduces the burn severity, e.g., [10,15,33,34]. Though the long term moderate cooling process is not sufficiently investigated by the international research community, scald burns should be treated by prolonged, i.e., at least 20–30 min, of tempered water application [33].

The significant influence of the epidermal thickness is also important and this result is in agreement with the findings by Ng and Chua [20] who showed that the thickness of the epidermis had a substantial influence on assessing burn thresholds. Spilling to clothing on the lap/thigh area exposes skin with fairly thin epidermis. The fact that this may result in superficial partial-thickness burns seems under-communicated to common people. That is strange, as this may happen in many daily life situations. Scalding when cooking food and when making hot beverages are representative examples. Spilling hot beverages in drive through arrangements where the car driver cannot easily remove the wet and hot fabric within a few seconds is another example. Scalding when stuck in the airplane seat during an accidental spill is yet another familiar scenario. A child, who has about 70% skin thickness compared to adults, is even more at risk of severe injury. This was particularly discussed in the recent study by Lau et al. [15] and Vallez et al. [34].

There are several uncertainties regarding the modelling in the present work. The thermal properties of skin and water, especially the thermal conductivity, changes with temperature. When spilling liquid to fabric, the spill does not necessarily supply just enough liquid to soak the fabric. If the fabric is spilled with hot liquid for e.g., a period of a few seconds, the assumption of an instant spill is not correct. Such a non-instant spill is indeed more realistic in an accidental spill. The continuous flow of liquid will then continue to heat the fabric, i.e., preventing the fabric temperature to drop as indicated in Figure 2. Since the injury development is very dependent on the wet fabric temperature, this will result in more severe skin injury. It is therefore advised to take the values from the present modelling as not more than just representative examples of minimum skin damage. Prolonged heating by spilling, e.g., 8 and 16 ounce (237 mL and 473 mL) beverage cups has been demonstrated by Abraham et al. [18] to give 1 mm deep burns at a water temperature of 82 °C.

The most important results in the present work are the benefit of lower liquid temperatures and fast removal of the fabric in scald scenarios. Should prompt fabric removal not be possible, immediate cooling through the clothing, e.g., by water or other liquids is beneficial [34].

Varying the parameters such as thermal conductivity and specific heat within reasonable limits did not change the main conclusions. The modeling therefore clearly demonstrates that prompt removal of the hot fabric is very important for skin injury mitigation.

## 5. Conclusions

In the present work, Pennes bioheat equation was solved numerically for hot beverage spills on clothing. Temperatures were analyzed both within the wetted fabric and the skin. The temperature development of the skin layers was presented and skin injury (damage integral) was calculated. Parameters influencing burn severity, such as clothing thickness, spilled liquid temperature, epidermis thickness, removal of wet and hot clothing and post scald water cooling were analyzed. The liquid temperature turned out to be the most important injury parameter, where liquid temperature of about 80–85 °C seems to be a limit for developing superficial partial-thickness burns in this minimum case where the liquid just wets the clothing. Spilling water in excess of just wetting the fabric, similarly severe burns will develop at even lower liquid temperatures due to the prolonged period of heat supply. Higher liquid temperatures, e.g., 90 °C, will nearly instantly develop severe burns. It was demonstrated that prompt removal of the fabric within the first seconds after the spill may significantly reduce skin injury. The general advice is therefore to avoid excessive heating of beverages and, if the liquid is spilled to the body, to quickly remove the wet any hot fabric. Prolonged tempered water cooling is advised to improve the post scald healing process.

## Figures and Tables

**Table 1:** Properties of the involved layers (thermal conductivity, k, density, ρ and specific heat, C, are from [11]). Base case (Case A) epidermis thickness is marked in bold face.

Skin Layer	k (W/m·K)	ρ (kg/m^3^)	$C_{P}$ (J/kg·K)	a (m^2^/s)	Thickness (m)
Epidermis	0.22	1200	3600	5.1 × 10^−8^	40, **50**, 60 & 80 × 10^−6^
Dermis	0.40	1200	3600	9.3 × 10^−8^	0.002
Sub cutaneous	0.20	1000	2500	8.0 × 10^−8^	0.010
Muscle	0.45	1000	3800	1.2 × 10^−7^	0.030

**Table 2:** Thermal properties of cotton fabric [29], water at representative 330 K [28] and estimated properties for soaked fabric.

Heat Source	k (W/m·K)	ρ (kg/m^3^)	C (J/kg·K)	ρC (J/m^3^·K)	Thickness (mm)
Dry cotton fabric	0.05	200	1340	2.7 × 10^5^	1.0
Water	0.65	1000	4184	4.2 × 10^6^	-
Soaked cotton fabric	0.65	1000	3470	3.6 × 10^6^	1.0

**Table 3:** Damage integral (Ω) for selected cases.

Case	$L_{\mathrm{epi}}$ (μm)	$L_{fabric}$ (mm)	$T_{W}$ (°C)	$t_{off}$ (s)	$t_{cool}$ (s)	Ω	Comments
A	50	1.0	85	-	-	0.973	Base case, 50 μm epidermis, fabric not removed
B	50	1.0	85	5.0	10	0.829	Fabric off at 5 s and water cooling at 10 s
C	50	1.0	85	2.5	5	0.475	Fabric off at 2.5 s and water cooling at 5 s
D	50	1.0	85	5.0	-	0.830	As Case B but no water cooling
E	50	1.0	85	2.5	-	0.475	As Case C but no water cooling
F	50	1.0	85	2.5	2.6	0.462	As Case C but water cooling at 2.6 s
G	40	1.0	85	-	-	1.209	As the base case but 40 μm epidermis
H	60	1.0	85	-	-	0.792	As the base case but 60 μm epidermis
I	80	1.0	85	-	-	0.542	As the base case but 80 μm epidermis
J	50	1.1	85	-	-	1.290	As the base case but 1.1 mm thick fabric
K	50	0.9	85	-	-	0.706	As the base case but 0.9 mm thick fabric
L	50	1.0	90	-	-	4.267	As the base case but spilled liquid at 90 °C
M	50	1.0	80	-	-	0.227	As the base case but spilled liquid at 80 °C
